# Effects of nanomicelle curcumin capsules on prevention and treatment of oral mucosits in patients under chemotherapy with or without head and neck radiotherapy: a randomized clinical trial

**DOI:** 10.1186/s12906-021-03400-4

**Published:** 2021-09-14

**Authors:** Seyed Javad Kia, Maryam Basirat, Hamid Saeidi Saedi, Seyed Ali Arab

**Affiliations:** 1 Dental sciences research center, Department of oral and maxillofacial medicine, School of dentistry, Guilan University of Medical Sciences, Rasht, Iran; 2 Department of radiation oncology, Cancer research center, Guilan university of medical sciences, Rasht, Iran; 3 Dental sciences research center, School of dentistry, Guilan University of Medical Sciences, Rasht, Iran

**Keywords:** Oral mucositis, Radiotherapy, Chemotherapy, Curcumin, Head and neck cancer, Cancer therapy

## Abstract

**Background:**

One of the most prevalent complications of chemotherapy and radiotherapy is oral mucositis (OM) and manifests as erythema and ulceration. Curcumin is one of the components of turmeric and possesses anti-inflammatory and anti-oxidative features. Some of studies have proved the effectiveness of Curcumin in OM. This study aimed to investigate the effects of nanomicelle Curcumin on OM related chemotherapy and head and neck radiotherapy.

**Methods:**

In this clinical trial study, 50 patients underwent chemotherapy with or without head and neck radiotherapy were divided into study and control group. The study group was received Curcumin nanomicelle capsules 80 mg twice a day and the control group took placebo two times a day for 7 weeks and the severity and pain of OM was measured.

**Results:**

Oral mucositis severity in control group in the first (*P* = 0.010), fourth (*P* = 0.022) and seventh (*P* < 0.001) weeks were significantly more than the study group. Pain grade in study group was lower than control group only in the seventh week. (*P* = 0.001) Additionally, NRS incremental gradient in control group was more than study group. OM severity in patients who underwent only chemotherapy in the control group were significantly more than the study group in all weeks. In patients who were under chemotherapy and head and neck radiotherapy, OM in control group was significantly more intense than the study group only in the fourth and seventh weeks.

**Conclusions:**

Nabomicelle Curcumin capsules is effective on prevention and treatment of head and neck radiotherapy and especially chemotherapy induced OM.

**Trial registration:**

Registered 12 February 2019 at Iranian Registry of Clinical Trials (IRCT). IRCT code: IRCT20100101002950N6. https://en.irct.ir/trial/36665. GUMS ethical code: IR.Gums.Rec.1397.296.

## Introduction

Chemotherapy and radiotherapy have been increasingly used as the main treatments for cancer. However, these therapeutic measures cause several complications, such as oral mucositis [1] Oral Mucositis (OM) is a condition manifested by erythema, edema, and ulceration of oral mucosa. Painful ulcerations can impair talking and swallowing in some patients, thereby impairing the quality of life and interfering with the treatment course [2].

The risk of OM development depends on factors including age, gender, poor oral hygiene, alcohol consumption, tobacco use, dosage and type of drugs used in chemotherapy, and radiotherapy dosage [3, 4].

Chemotherapy-induced OM is caused by the early release of inflammatory cytokines and Reactive Oxygen Species (ROS), decreased Keratinocyte Growth Factor (KGF), activation of transcription factors such as NF-Kβ, and increased apoptosis in the mucosa [2, 5].

Radiotherapy-induced OM is caused by injury to arterioles and salivary glands, the release of inflammatory cytokines and ROS, and provoking different reactions leading to apoptosis of basal cells and mucosal inflammation [6, 7].

40% of patients undergoing standard chemotherapy, 30–60% of patients under head and neck radiotherapy, and 90% of patients receiving both chemotherapy and head and neck radiotherapy develop OM [8]. Chemotherapy-induced OM is milder and tends to heal faster than the OM induced by head and neck radiotherapy [9].

Clinical management of radiotherapy- and chemotherapy-induced OM include maintaining oral hygiene, physical therapy (e.g., cryotherapy), and medications including antibacterial agents (e.g. chlorhexidine), anti-inflammatory agents (e.g., benzydamine), cytoprotective agents (e.g. amifostine), biologic response modifiers (e.g. G-CSF), local anesthetic agents, and analgesics. However, according to different studies, OM treatment is controversial, and these therapeutic modalities are supportive treatments and not approved therapeutic and preventive measures [10–12].

*C. longa* (turmeric) is a medicinal plant that has been used in the traditional medicine of China and Southeast Asia. The three curcuminoids, including curcumin, demethoxycurcumin, and bisdemethoxycurcumin are the active ingredients in turmeric. Curcumin (1, 7-bis (4-hydroxy-3-methoxyphenyl) hepta-1, 6-diene-3, 5-dione) or diferuloylmethane is a natural component produced in the rhizomes of *C. longa* and other species of genus Curcuma [13, 14]. This substance is believed to be responsible for the anti-inflammatory and antioxidant properties of turmeric, including regulation of cell growth and apoptosis [14].

TNF-α and NF-Kβ are two important factors playing a role in several inflammatory disorders. Curcumin exerts its anti-inflammatory effects through reducing TNF-α and NF-Kβ and suppressing the post-inflammatory pathways [15, 16].

It is believed that curcumin exerts its antioxidant effects through increasing the plasma levels of superoxide dismutase (SOD) and glutathione peroxidase (GSH), increasing catalase activity, and decreasing plasma levels of lipid peroxidase. These alterations can suppress oxidative stress and contribute to the anti-inflammatory properties of curcumin [17].

Concentration of Curcumin in blood is very low after oral administration of curcumin has insufficient therapeutic effects due to low blood level and absorption, chemical instability and rapid systemic elimination. Oral administration of curcumin does not cause sufficient therapeutic; because there are extremely low blood levels of curcumin following oral consumption due to insufficient absorption, chemical instability, and rapid systemic elimination [18] In vivo study showed that low-dose (20 mg/kg) Nano-Curcumin has an equivalent therapeutic effect as high-dose (400 mg/kg) pure Curcumin [19].

Various efforts have been made to increase the curcumin bioavailability and resistance to metabolic processes in order to increase its serum levels. These efforts include using different methods and materials such as administration with adjuvants (piperine), bio-conjugates [turmeric oil, glycine, alanine, and epigallocatechin-3-gallate (EGCG)), and lipids (phospholipids), encapsulation into nanoparticles (liposomes, micelles, noisome, nanogels, chitosan, gold, silver, cyclodextrin, dendrimer, solid lipids), and use of proteins (BSA, soy protein isolate) and other methods (hyaluronic acid, hydrogel, polymer, PEG-PEI, emulsification, beta-lactoglobulin) [20, 21].

Encapsulation into nanoparticles can increase the bio-availability and solubility of lipophilic substances such as curcumin, thereby leading to improved therapeutic effects, controlled release rate, decreased toxicity, long-term presence in circulation, and modified pharmacokinetic profile. Nanomicelles are an example of these nanoparticles [22].

The core-shell structure of nanomicelle protects the inner core from water, so it can be an appropriate alternative to deliver curcumin. Also, these curcumin nanomicelles have other advantages, including affordable costs, easy development, resistance to degradation, and improved solubility, bioavailability, and stability [23, 24].

Regarding the anti-inflammatory and antioxidant effects of curcumin, the effectiveness of topical curcumin in alleviating the OM induced by chemotherapy and radiotherapy has been shown in some studies [25–29]. Furthermore, 1 g curcumin capsules have proved to be effective in alleviating the OM induced by head and neck radiotherapy [30] 80 mg SinaCurcumin in the form of nanomicelle also was evaluated and it was curative for OM induced by head and neck radiotherapy [31]. Because of anti-inflammatory and anti-oxidative characteristics of Curcumin, we expected that it would be an appropriate treatment for oral mucositis induced by chemotherapy and head and neck radiotherapy. To date, no randomized clinical trial has evaluated the effectiveness of curcumin in the form of nanomicelles in patients with chemotherapy who manifest OM. Therefore, the present study was conducted to investigate the effectiveness of capsules containing curcumin nanomicelles in patients undergoing chemotherapy with or without head and neck radiotherapy who have also developed oral mucositis.

## Methods and materials

The current RCT report followed the standard checklist of CONSORT. The present study is a parallel double-blinded randomized clinical trial performed at cancer center of Razi hospital, Rasht, Iran.

### Participants

Two groups of patients were entered into the study; first, the patients who were under chemotherapy and head and neck radiotherapy including patients who have head and neck cancer; second, patients who only underwent chemotherapy due to cancer in any other organs. The chemotherapy drugs used for the patients were Cisplatine 30-50 mg and 5FU 640–750 mg and the radiotherapy dosage was 6000–7000 cGy. All the patients were entered into the study before the start of cancer treatment. Onset of the study was coincided with the beginning of cancer therapy.

The exclusion criteria included the patients with oral lesions unrelated to the treatment or malignancy, individuals with systemic or immunosuppressive diseases commonly manifesting with oral lesions, being allergic to turmeric, smokers, pregnant women, taking medications that could induce oral lesions, receiving anticoagulants and patients who were unable to take the capsules. At the baseline, patients had no oral lesion unrelated to cancer in both study and control groups.

The patients were randomly divided into two study and control groups.

To calculate the sample size, considering measurement in two groups, distribution of two groups must be determined. Therefore, the formula for differences in two population and pain score variable was used. According to a statistical power of 95%, an error level of 0.05, and a standard deviation of 2.1 (study group) and 2 (control group) and d = 2.5 which resulted from previous studies and calculating 20% of exclusion, the minimum sample size was 22 people for each group [28]. To improve the power of study we use 25 participants in the study. In the formula d is the mean difference and f is the follow rate.

$$ {\displaystyle \begin{array}{l}{n}_0=\frac{{\left({z}_{1-\frac{\alpha }{2}}+z{}_{1-\beta}\right)}^2\left({\sigma}_1^2+{\sigma}_2^2\right)}{(d)^2}=\frac{{\left(1.96+1.64\right)}^2\left({2.1}^2+{2}^2\right)}{(2.5)^2}=17.44\\ {}n={n}_0\times \frac{1}{1-f}\cong 22\end{array}} $$

### Study group

25 patients in the study group were received 80 mg nanomicelle Curcumin capsules twice a day after food consumption. The nanomicelle capsules were made by ExirNanoSina Company. Nanomicelle Curcumin capsule is red soft gel which is available as SinaCurcumin in Iran.

### Control group

25 patients in the control group were taken placebo capsules twice a day after food. The placebo capsules were mostly made of sugar and manufactured by Sobhan Daru CO (Sobhan Darou Co, Tehran, Iran). It was a red soft gel capsule exactly the same as SinaCurcumin apparently.

#### Interventions, randomization and blinding

Patients who meet the inclusion criteria were referred from oncology private offices in Rasht to cancer center of Razi hospital. Patients were examined clinically at the onset of study by an oral and maxillofacial medicine specialist who was not involved in the study to confirm that they do not have any oral inflammatory or infectious lesion and do not meet the exclusion criteria. Demographic data of the study such as age, gender, smoking habit and medical history and type and dose of chemotherapy drug and dose of radiotherapy were also recorded via a checklist. Blocking randomization was used in this study. For random allocation, patients were given a number from 1 to 50; then using SAS 9.1 software were randomly divided into 2 groups and each participant was given “a” or “b” letter. Afterward, patients with “a” letter were placed in the control and patients with “b” letter were entered to the study groups. The placebo and SinaCurcumin capsules were same in the state of color, shape and size and were put in identical boxes for each patient and they were blinded to researchers and patients. Random allocation sequence and distribution of capsules were generated by a person who was not involved in the study and they were concealed from researcher. The onset of study was coincided with the start of patients’ cancer therapy. Additionally, all patients were advised to brush and wash their mouth three times a day.

The treatment duration was 7 weeks and the follow up sessions were first, second, fourth and seventh weeks. The patient’s clinical examination, besides the measurement of their lesions and pain severity, was performed by an oral medicine specialist without knowing the grouping of patients. The WHO Mucositis Scale was used to assess the OM severity. This scale was graded as follows:

0) No lesion was seen
1) (mild) oral soreness, erythema
2) (moderate) oral erythema, ulcers, solid diet tolerated
3) (severe) oral ulcers, liquid diet only
4) (life-threatening) oral alimentation impossible

The pain scores were recorded using the 10-point Numerical Rating Scale (NRS), in which score 0 means “no pain” while score 10 means “the worst pain possible”.

The patients were informed of the study design and plans and informed consent ​​​​was obtained from all the patients. The consent form and all experimental protocols were approved by the Ethics Committee of the Guilan University of Medical Sciences (GUMS ethical code: IR.Gums.Rec.1397.296). The patients were recommended to use Lidocaine mouthwash in case of intolerable pain before food consumption. The registration number of clinical trial was in a primary registry in the WHO registry network was IRCT20100101002950N6 and all methods were performed in accordance with the relevant guidelines and regulations.

#### Statistical analysis

To determine the normality of data, Kolmogorov–Smirnov test was used. All results were reported as frequency and mean and standard deviation (mean ± *SD*). Repeated measure analysis of variance was used to assess the time-dependent changes in OM severity and pain within the period of study in two groups. The differences of OM severity and pain grade between two groups in each time were analyzed by Independent T-test. The posthoc Bonferroni test was used to determine differences of OM severity and pain grade within groups in different times. All The statistical analysis was performed using the SPSS 22 software.

## Results

From 67 patients primarily were entered to the study, 17 participants were excluded. 3 patients were declined to participate, 4 had oral lesion at the baseline, 7 were smokers and 3 had immunosuppressive diseases. 50 patients undergoing chemotherapy with or without head and neck radiotherapy were analzed in the study, from whom 37 were under only chemotherapy, and 13 were receiving both chemotherapy and head and neck radiotherapy. The control and study group were followed at first, second, fourth and seventh weeks.

The study included 28 male and 22 female patients and a mean age of 55.96 ± 1.10 (54.98 and 56.94 for the study group and control group, respectively). The study group (15 male and 10 female) received capsules of curcumin nanomicelles to prevent and minimize the OM, while the control group (14 male and 11 female) received placebo capsules.

The groups were not significantly different in terms of age, gender, type and dosage of chemotherapy, radiotherapy dosage, tumor type, and tumor location (Table 1). The mean scores of erythema and ulceration assessed using the WHO Mucositis Scale were not significantly different between the patients older than 56 and those who were 56 or younger in the 7-week study duration (*p* = 0.193). Also, The OM severity was not significantly different between male and female patients (*p* = 0.316).

**Table 1:** Patients information

Variable	Study	Control	Significance (Chi-Square standardized statistic)
**Sexuality**			**0.569 (0.32)**
Male	15 (60%)	13 (52%)	
Female	10 (40%)	12 (48%)	
**Age**			**0.396 (0.72)**
≤ 56	14 (56%)	11 (44%)	
> 56	11 (44)	14 (56%)	
**Tumor type**			**0.994 (0.34)**
SCC	5 (20%)	5 (20%)	
Lymphoma	3 (12%)	3 (12%)	
Adenocarcinoma	9 (36%)	7 (28%)	
Liemiosarcoma	1 (4%)	2 (8%)	
Hepatocellular carcinoma	1 (4%)	1 (4%)	
Angiosarcoma	1 (4%)	–	
breast Ductal carcinoma	2 (8%)	3 (12%)	
breast Lobular carcinoma	1 (4%)	1 (4%)	
Ovarian carcinoma	2 (8%)	3 (12%)	
**Tumor location**			**0.951 (0.05)**
Rectum	7 (28%)	6 (24%)	
Ovary	2 (8%)	3 (12%)	
Gastric	3 (12%)	3 (12%)	
Breast	3 (12%)	4 (16%)	
Liver	2 (8%)	1 (4%)	
Colon	1 (4%)	2 (8%)	
Larynx	2 (8%)	3 (12%)	
Hypo-pharynx	2 (8%)	2 (8%)	
Palate	2 (8%)	1 (4%)	
Lymphoma in neck	1 (4%)	–	
**Chemotherapy drug and dosage**			**0.999 (0.23)**
Cisplatin 30	2 (11.1%)	1 (5.3%)	
Cisplatin 50	8 (44.4%)	9 (47.3%)	
5FU 640	5 (27.7%)	6 (31.6%)	
5FU 700	1 (5.6%)	2 (10.5%)	
5FU 750	2 (11.1%)	1 (5.3%)	
**Radiotherapy dosage**			**0.095**
6000 cGy	5 (71.5%)	1 (16.7%)	
6600 cGy	0	1 (16.7%)	
7000 cGy	2 (28.5%)	4 (66.6%)	

**Fig. 1:**
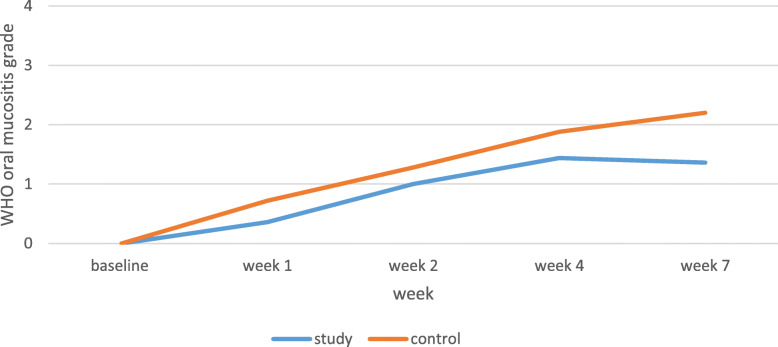
Oral mucositis severity in patients undergoing chemotherapy with or without head and neck radiotherapy

The OM was significantly more severe in the control group than the study group in weeks 1 (*P* = 0.010), 4 (*P* = 0.022), and 7 (*P* < 0.001), and the incremental gradient was higher in the control group than the study group. OM severity mean scores of the control group were 0.72, 1.88, and 2.2 in weeks 1, 4, and 7, respectively, with a gradual increase in 7 weeks (*P* < 0.001). The OM severity alterations in the study group were significantly increasing from the baseline assessment to week 4 (P < 0.001) while insignificantly decreasing from weeks 4 to 7. Mean scores of erythema and ulceration of the study group were 0.36, 1.44, and 1.36 in weeks 1, 4, and 7, respectively (Table 2, Fig. 1).

**Table 2:** OM severity and pain grade in patients under chemotherapy with or without head and neck radiotherapy

	WHO oral mucositis grade mean in study group	WHO^b^ oral mucositis grade mean in control group	Significance (T-test statistic)	NRS^a^ mean in study group	NRS mean in control group	Significance (T-test statistic)
Participants	25	25	–	25	25	–
Base line	0	0	–	0	0	–
Week 1	0.36 ± 0.49	0.72 ± 0.46	0.010 (2.68)	0.68 ± 1.11	0.64 ± 1.03	0.896 (0.13)
Week 2	1 ± 0.64	1.28 ± 0.46	0.083 (1.77)	1.36 ± 1.91	1.84 ± 1.57	0.337 (0.97)
Week 4	1.44 ± 0.58	1.88 ± 0.73	0.022 (2.36)	2.44 ± 2.00	3.28 ± 1.57	0.105 (1.65)
Week 7	1.36 ± 0.64	2.20 ± 0.71	< 0.001 (4.41)	2.64 ± 2.04	4.44 ± 1.68	0.001 (3.40)

^a^Numerical rating scale

^b^World Health organization

**Fig. 2:**
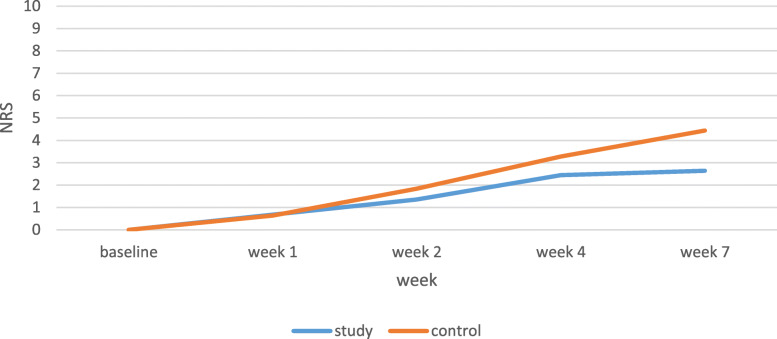
Pain score in patients under chemotherapy with or without head and neck radiotherapy

The pain score was lower in the study group than the control group in week 7 (*P* = 0.001). Additionally, NRS incremental gradient was higher in the control group than the study group. The mean pain score significantly increased throughout the 7 weeks in both groups (*P* < 0.001). NRS mean scores of the control group were 0.64 and 4.44 in weeks 1 and 7, respectively, while the NRS mean scores of the study group were 0.68 and 2.64 in weeks 1 and 7, respectively (Table 2, Fig. 2).

**Fig. 3:**
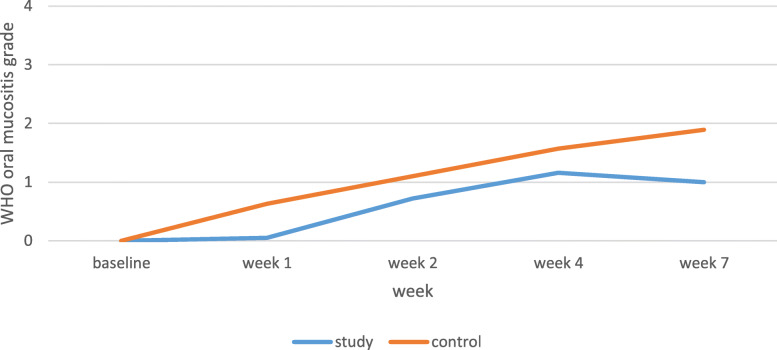
Oral mucositis severity in patients undergoing chemotherapy without head and neck radiotherap

The OM severity was significantly lower in the patients undergoing only chemotherapy in the study group than the control group in all the weeks (*P* < 0.001). Also, the changes were incremental in both groups in the 7-week period (Table 3, Fig. 3).

**Table 3:** OM severity and pain grade in patients under chemotherapy without head and neck radiotherap

	WHO oral mucositis grade mean in study group	WHO oral mucositis grade mean in control group	Significance (T-test statistic)	NRS mean in study group	NRS mean in control group	Significance (T-test statistic)
Participants	18	19		18	19	
Base line	0	0	–	0	0	–
Week 1	0.05 ± 0.23	0.63 ± 0.49	< 0.001 (0.576)	0.05 ± 0.23	0.26 ± 0.65	0.207 (0.20)
Week 2	0.72 ± 0.46	1.10 ± 0.31	0.006 (0.383)	0.27 ± 0.66	1.21 ± 1.08	0.004 (0.93)
Week 4	1.16 ± 0.38	1.57 ± 0.50	0.008 (0.412)	1.44 ± 1.24	2.52 ± 0.96	0.005 (1.08)
Week 7	1.00 ± 0.48	1.89 ± 0.45	< 0.001 (0.894)	1.61 ± 1.28	3.84 ± 1.16	< 0.001 (2.23)

**Fig. 4:**
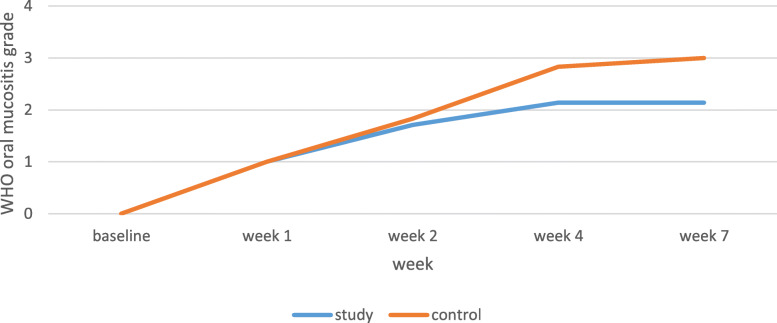
Oral mucositis severity in patients undergoing chemotherapy with head and neck radiotherapy

The OM severity was significantly lower in the patients undergoing both chemotherapy and head and neck radiotherapy in the study group than the control group in weeks 4 (*P* = 0.009) and 7 (*P* = 0.012) (Table 4, Fig. 4).

**Table 4:** OM severity in patients under chemotherapy with head and neck radiotherapy

	WHO oral mucositis grade mean in study group	WHO oral mucositis grade mean in control group	Significance (T-test statistic)	NRS mean in study group	NRS mean in control group	Significance (T-test statistic)
Participants	7	6		7	6	
Base line	0	0	–	0	0	–
Week 1	1.00	1.00	–	2.28 ± 0.75	1.83 ± 1.16	0.309 (0.88)
Week 2	1.71 ± 0.48	1.83 ± 0.40	0.646 (0.119)	4.14 ± 0.89	3.83 ± 1.16	0.113 (0.67)
Week 4	2.14 ± 0.37	2.83 ± 0.40	0.009 (0.690)	5.00 ± 1.00	5.33 ± 1.21	0.805 (0.15)
Week 7	2.14 ± 0.37	3.00 ± 0.63	0.012 (0.857)	5.28 ± 0.75	6.16 ± 2.13	0.238 (0.52)

**Fig. 5:**
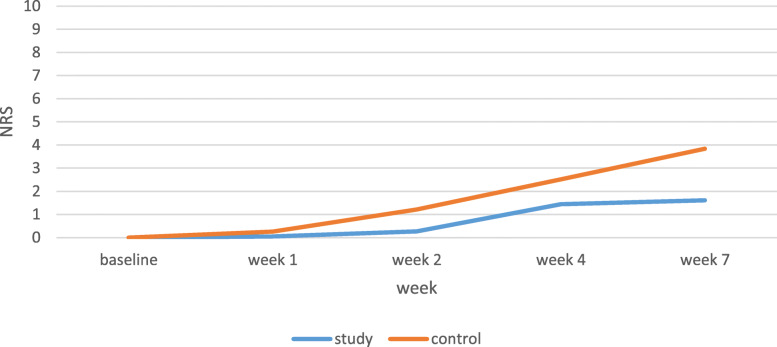
Pain score in patients undergoing chemotherapy without head and neck radiotherapy

**Fig. 6:**
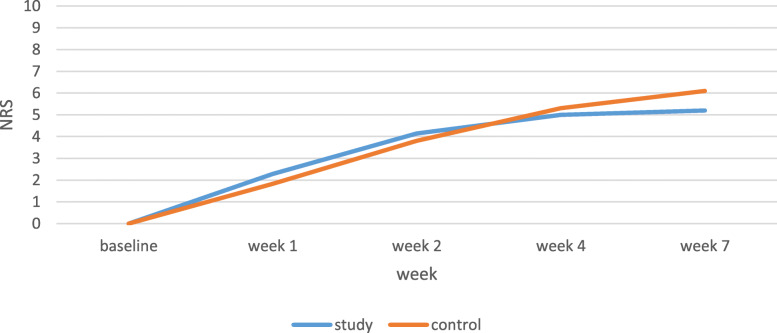
Pain score in patients undergoing chemotherapy with head and neck radiotherapy

Although differences in NRS mean scores in patients who were only under chemotherapy were significant (*P* < 0.001) between two groups within time, they were not significant (*P* = 0.128) in patients receiving both chemotherapy and radiotherapy. (Table 4, Figs. 5 and 6).

## Discussion

Regarding the emergence of novel therapeutic modalities, oral mucositis has become an important complication of cancer treatment. There are different medications and methods to alleviate the OM, including sodium carbonate, local anesthetics, diphenhydramine, chlorhexidine, morphine (2% mouthwash), doxepin, anti-inflammatory agents such as benzydamine hydrochloride mouthwash, and local steroids. Most of these therapeutic methods are palliative or need more evidence to prove their effectiveness in the prevention and reduction of OM symptoms. In the present study, we used the therapeutic protocol of capsules containing 80 mg curcumin in the form of nanomicelles twice a day for 7 weeks.

The mean OM score was not significantly different between the patients older than 56 and those who were 65 or younger in 7 weeks, which was compatible with the study by Al-Ibrahimi et al. (2016) that showed OM was associated with age [32]. However, Damascena et al. (2018) evaluated the effective factors in OM development during chemotherapy and found that age was positively correlated with OM severity [33]. These different findings regarding the association of age with OM severity can be explained by differences in study populations, sample sizes, disease severity, and chemotherapy dosage.

The mean scores of erythema and ulceration in the study were not significantly different between the genders in 7 weeks. However, Nishi et al. (2019) investigated the factors associated with severe oral mucositis in patients undergoing head and neck radiotherapy and found out that OM was more common in male than female patients [34]. This controversy can be explained by the differences in the study population, sample size, type and dosage of chemotherapy, location of the lesion, and the general and nutritional condition of the patients.

In the present study, erythema and ulceration scores based on the WHO Mucositis Scale showed that the OM severity alterations were significantly increasing in the study group from baseline assessments to week 4, while insignificantly decreasing for week 4 to week 7. However, the OM severity alterations were significantly increasing in the study duration in the control group, and the incremental gradient was higher in the control group than the study group. The WHO Mucositis Scale scores were higher in the control group in the study group in weeks 1, 4, and 7. Delavarian et al. (2019) studied the effect of capsules containing 80 mg curcumin in the form of nanomicelles on patients with OM due to head and neck radiotherapy in 6 weeks and found out that the OM was milder in the study group than in the control group, which is compatible with our study [31]. Moreover, Elad et al. (2013) investigated curcumin in a typical form for OM prevention on pediatric patients and found out that 4 of 5 patients developed OM, but it was relatively mild [25]. Mansurian et al. (2015) investigated the effect of *C. longa* topical gel on radiotherapy-induced OM in patients suffering from head and neck malignancies and showed its effectiveness in reducing the OM symptoms [28]. A study by Charantinath et al. (2016) evaluated the effect of curcumin on the OM induced by radiochemotherapy and showed that curcumin gel could be an effective and safe alternative medication in the OM treatment [27]. Patil et al. (2015) evaluated the effect of curcumin mouthwash in the OM induced by radiochemotherapy and found out that it was more effective in accelerating the healing process than the chlorhexidine mouthwash [29]. In general, the results from different studies are compatible with the present study in the effectiveness of curcumin in the prevention and treatment of radiochemotherapy-induced OM, showing the decreased severity, decelerated progression, and accelerated healing of OM by anti-inflammatory, antioxidant, and anti-microbial features of curcumin. However, the yellow color of curcumin as a topical treatment is not acceptable for many patients, which is one of the limitations of this treatment. Furthermore, there are studies about the effect of systemic curcumin on reducing articular inflammation, and it seems that curcumin can improve oral hygiene and the ability to open the mouth, thereby facilitating the OM treatment.

The pain scores (NRS) increased in both groups during the study duration, with the incremental gradient being higher in the control group than the study group. Patients undergoing chemotherapy and radiotherapy, in particular, experience different complications such as xerostomia, impaired perfusion of oral mucosa and muscles, reduced mouth opening, TMJ impairment, and inability to maintain oral hygiene [35]. Poor oral hygiene, inappropriate nutrition, and persistent liquid consumption lead to dental problems and subsequent pain. Additionally, the adverse effects of radiotherapy and chemotherapy usually develop after the first week and exacerbate in the last weeks due to higher infection susceptibility and pain because of cytopenia. However, mean pain scores were lower in the study group than the control group in 7 weeks, with a significant inter-group difference in week 7. This finding was compatible with the study by Charantinath (2016) on the effects of curcumin topical gel on the OM [27].

The OM of the patients undergoing only chemotherapy was significantly more severe in the control group than the study group in all weeks, and the scores of the WHO Mucositis Scale increased in both groups in the study duration. Also, the OM severity in the patients under both chemotherapy and head and neck radiotherapy was significantly higher in the control group than the study group in weeks 4 and 7. According to the mentioned results, curcumin is more efficient in the OM treatment and prevention in patients under just chemotherapy than the patients receiving both chemotherapy and head and neck radiotherapy. According to the studies, OM is more common and severe in patients undergoing head and neck radiotherapy than those receiving only chemotherapy [11, 12]. Head and neck radiotherapy leads to the release of reactive oxygen species that subsequently cause cellular and DNA damage in the basal epithelium and sub-mucosa. These events activate a chain of reactions leading to the production of pro-inflammatory cytokines, basal cell apoptosis, and mucosal lesions. The same process happens in the oral mucosa during chemotherapy [6–9]. Head and neck radiotherapy also affects the salivary glands by capillary and arteriole destruction, leading to atrophy, fibrosis, and degeneration of the salivary glands, so xerostomia can be a chief complaint of such patients, exacerbating the OM process [36]. However, the effect of chemotherapy on the salivary glands is controversial [37–39]. Curcumin is an anti-inflammatory and anti-oxidative drug; therefore, it only influences on inflammatory pathway of OM pathophysiology and is ineffective on salivary glands destruction and xerostomia. In conclusion, the results indicated that curcumin had different effects on patients under chemotherapy and head and neck radiotherapy. Additionally, 80 mg curcumin capsules are not toxic (toxicity dose is 12 g) [40].

Number of patients was a challenging part of the study. Although the patients were homogenous in both groups, it is better to make patients more specific in further studies. In other words, location of tumor, type of drug and treatment strategy should be more restricted.

Another limitation is serology assessment of patients. For some reasons we could not access to laboratory tests of the patients. Further studies can analyze serologic inflammatory factors.

## Conclusion

Capsules of curcumin nanomicelles were effective in preventing and treating radiotherapy and chemotherapy-induced OM and can be an acceptable alternative for the current palliative and local treatments. It is more effective in chemotherapy induced OM rather than head and neck radiotherapy related OM. However, according to our findings and Delavarian’s study, there is no significant difference in the OM severity with increased doses (more than 1 capsules a day).

## Data Availability

The datasets used and analyzed during the current study are not publicly available due to further studies related to the data but are available from the corresponding author on reasonable request.

## Abbreviations

OM: Oral Mucositis
WHO: World Health Organization
TNF: Tumor Necrosis Factor
ROS: Reactive Oxygen Species
KGF: Keratinocyte Growth Factor
NF-Kβ: Nuclear Factor- Kappa B
NRS: Numeric Rating Scale
